# Chronic Rheumatism and Rheumatoid Arthritis

**Published:** 1908-03-07

**Authors:** J. Lindsay


### The General Practitioner's Column.

[Contributions to this Column are invited, and if accepted will be paid for.]

### CHRONIC RHEUMATISM AND RHEUMATOID ARTHRITIS.

### TREATED BY SALICYLATE OF COLCHICINE.

By J. LINDSAY, M.D., Ch.B.

The treatment by drugs of chronic rheumatism and rheumatoid arthritis still remains in a very unsatisfactory state. Among the drugs which are at present in most favour may be mentioned salol and $\beta$ naphthol, while guaiacol and guaiacol carbonate are held to be especially indicated in the more subacute forms of those conditions.

While the influence of colchicum in the above conditions is by no means so beneficial as in gout, yet the close affinity of the rheumatic and gouty states would in itself suggest its employment. Numbers of cases of so-called chronic rheumatism are, no doubt, allied to the gouty diathesis; and some cases which defy a definite diagnosis, may both, from a clinical and therapeutical standpoint, be classed under the old term of "rheumatic gout." That colchicum had a beneficial action as a pain-reliever, not only in gout but also in chronic rheumatism, was recognised early by Trousseau and others; but it was observed that in order to obtain the desired effects such doses had to be administered as to bring on a considerable degree of cardio-vascular depression; and these toxic effects were largely accountable for the ultimate restriction in its use.

The salicylate of colchicine having been tried with satisfactory results in cases of chronic gout, bringing about relief of pain without any of the depressant effects, it was considered advisable to treat some cases of chronic rheumatism and rheumatoid arthritis with the view, if possible, of relieving the joint pains. The following brief notes on four cases serve to illustrate its beneficial action:—

Case 1.—G. L., a railway porter, forty years of age, complained of pain and stiffness in both knees, ankles, shoulders, elbows, and wrists. His proximal inter-phalangeal joints were spindle-shaped: there was a history of onset in the ankles fourteen years before, ultimately affecting, and, to some extent crippling, all joints. The present attack began three months ago, very severely in the shoulders, knees, and ankles, especially at night after getting into bed. The patient had never had acute rheumatism, and there was no cardiac murmur. He was given salicylate of colchicine, gr. 1-32 ter in die. In two or three days the pains were much diminished, and at the end of a week's time he was quite free from joint pain. The drug was continued twice daily for another week, by the end of which time there had been no return of the pain.

Case 2.—L. R., a married woman, thirty-five years of age. A case of chronic rheumatism, affecting all the larger joints, more particularly the knees and ankles, with a history of rheumatic pains during last two years, but none of acute rheumatism. When first seen she was unable to walk a step, having to be moved from place to place in a wheel-chair. Salicylate of colchicine, gr. 1-32, was administered twice daily. Rapid improvement took place, and in a few days the constant dull, aching pains in the knees and ankles had almost gone, movement at these joints was permitted, and at the end of three weeks, she was able to walk upstairs with only slight assistance.

Case 3.—A. H., a married woman, twenty-one years of age. A case of rheumatoid arthritis following on childbirth. The knees, ankles, elbows, wrists, finger-joints, and temporo-maxillary joints were all affected, being swollen and painful. When seen, about two months after the onset of the initial symptoms, the temperature was rising each evening to about 101 degs. The patient was confined to bed, any attempt at movement of joints giving rise to much pain. She took salicylate of colchicine gr. 1-32 ter in die. From the first improvement was noticeable. The pain was much less, and the evening temperature in a week's time was steady at normal. Two weeks later the patient was up and getting about with the aid of crutches.

Case 4.—M. R., a married woman, forty-three years of age. A case of chronic rheumatoid arthritis. She complained of pain, stiffness, and deformity of joints, the onset of which was eight years previously in the shoulders and knees. The finger-joints, elbows, wrists, and ankles were subsequently affected. Salicylate of colchicine, gr. 1-32, was prescribed twice daily. Improvement rapidly took place, much relief being afforded from the joint pains. Patient who, when first seen, was unable to raise her arm above her shoulder, was, at the end of four weeks, able to do up her hair and move about with comparative ease and comfort.

From these cases, which are in no way specially selected, it will be admitted that salicylate of colchicine is a drug worthy of an extended trial by the profession.

Salicylate of colchicine will not in itself cure either chronic rheumatism or rheumatoid arthritis, but its use will to some extent give the patient that which he most desires, namely, some relief from the joint-pains. It is, of course, needless to observe that all the recognised forms of treatment, including dieting, hydro-therapy, etc., should be carried on concurrently, and to these the employment of salicylate of colchicine should only be looked on as a highly beneficial adjunct.

---

[The following information about colchicine salicylate is extracted from the recently-published British Pharmaceutical Codex:—

" . . . . A yellow amorphous powder, readily soluble in water, alcohol, and ether. Colchicine salicylate may be made into pills with sugar of milk and glycerin of tragacanth, but is more generally dispensed in capsules containing . . . . colchicine salicylate dissolved in methyl salicylate. Colchicine salicylate should be kept in a dark place."—Ed.]

